# Arginine: New Insights into Growth Performance and Urinary Metabolomic Profiles of Rats

**DOI:** 10.3390/molecules21091142

**Published:** 2016-08-29

**Authors:** Guangmang Liu, Xianjian Wu, Gang Jia, Xiaoling Chen, Hua Zhao, Jing Wang, Caimei Wu, Jingyi Cai

**Affiliations:** 1 Institute of Animal Nutrition, Sichuan Agricultural University, Chengdu 611130, Sichuan, China; wuxianjianqq@hotmail.com (X.W.); jiagang700510@163.com (G.J.); xlchen@sicau.edu.cn (X.C.); zhua666@126.com (H.Z.); zhuomuniao278@163.com (C.W.); jycai2004@aliyun.com (J.C.); 2 Key Laboratory for Animal Disease-Resistance Nutrition of China Ministry of Education, Chengdu 611130, Sichuan, China; 3 Maize Research Institute, Sichuan Agricultural University, Chengdu 611130, Sichuan, China; wangj221@gmail.com

**Keywords:** arginine, metabolism, metabolomic profiles, urine

## Abstract

Arginine regulates growth performance, nutrient metabolism and health effects, but the underlying mechanism remains unknown. This study aims to investigate the effect of dietary arginine supplementation on rat growth performance and urinary metabolome through ^1^H-NMR spectroscopy. Twenty rats were randomly assigned to two groups supplemented with 0% or 1.0% l-arginine for 4 weeks. Urine samples were analyzed through NMR-based metabolomics. Arginine supplementation significantly increased the urine levels of 4-aminohippurate, acetate, creatine, creatinine, ethanolamine, formate, hippurate, homogentisate, indoxyl sulfate, and phenylacetyglycine. Conversely, arginine decreased the urine levels of acetamide, β-glucose, cirtulline, ethanol, glycine, isobutyrate, lactate, malonate, methymalonate, *N*-acetylglutamate, *N*-methylnicotinamide, and propionate. Results suggested that arginine can alter common systemic metabolic processes, including energy metabolism, amino acid metabolism, and gut microbiota metabolism. Moreover, the results also imply a possible physiological role of the metabolism in mediating the arginine supplementation-supported growth of rats.

## 1. Introduction

Arginine is a nutritionally essential amino acid for young mammals, particularly under stressful conditions [1,2], and exhibits versatile functions in metabolism and physiology. Arginine is an important component of multiple systems, such as the circulatory, gastrointestinal, immune, and reproductive systems in humans and animals [3,4]. Several studies have indicated that arginine is an allosteric activator of *N*-acetylglutamate synthase, which converts glutamate and acetyl-CoA into *N*-acetylglutamate; the converted enzyme is also an essential allosteric activator of carbamoylphosphate synthase I, a key enzyme in the hepatic urea cycle for ammonia detoxification [5]. As such, arginine is necessary in activating hepatic urea synthesis.

Arginine stimulates the secretion of growth hormones and insulin in mammals and thus regulates protein metabolism [6]. Arginine activates the mammalian targets of the rapamycin cell signaling pathway [7,8], which is important in protein synthesis, cell growth, and cytoskeletal remodeling of various tissues, such as skeletal muscles [9]. Arginine also facilitates wound healing and enhances intestinal epithelial cell proliferation and migration; these processes are associated with the repair of damaged intestinal villi [10,11]. Arginine supplementation to corn- and soybean meal-based diets of 110 d-old barrows for 60 d decreased serum triglyceride levels and reduced fat accretion in white adipose tissues of obese rats [12], obese humans [13], obese sheep [4], and growing-finishing pigs [14]. Moreover, arginine induces lipolysis and reduces lipogenesis in white adipose tissues, as well as enhances the oxidation of fatty acids and glucose by skeletal muscles [14,15]. Dietary arginine supplementation also increases glycogen content but decreases lactate content in skeletal muscles as a result of reduced glycogenolysis and glycolysis [16]. Furthermore, arginine regulates mammalian embryonic survival and growth [4,17,18] and plays an important role in the immune system. Arginine stimulates the functional activities of different cell types, including natural killer cells, macrophages, lymphokine-activated killer cells, T cells, and B cells in the immune system [19,20,21]. Zhu et al. reported that l-arginine regulated intestinal mucosal immune barrier function in weaned pigs after *Escherichia coli* LPS challenge [22].

Arginine is the precursor for the synthesis of several biologically active molecules, including nitric oxide (NO), ornithine, polyamines (putrescine, spermidine, and spermine), creatine, and agmatine [23]. Polyamines are essential for the proliferation and differentiation of cells, such as intestinal cells and lymphocytes [24], and modulation of the plasma and ileum metabolome in rats [25,26,27]. NO regulates ovulation and placental growth, as well as viral and bacterial infections. Urine is the most readily acquired biofluid. The collection of urine are more noninvasive than that of blood. The effects of nutrients in the metabolic profiles are more obvious in the urine than in the plasma [28]. The chemical components of urine, directly affected by the functions of different body systems, are good indicators of metabolic status and can reveal key information about animal’s health. Recent study demonstrated that arginine significantly perturbed the plasma metabolite levels of amino acids, lipids, and lactate in pigs [29,30], and altered urine and plasma metabolome under oxidative stress [31]. However, there is no information about the urinary metabolite profiles of arginine supplementation in any mammalian in vivo system under non-oxidative stress, which is valuable for investigation. Metabolomics provides a novel strategy to determine changes in metabolic endpoints of physiological modulation processes of an organism after specific nutritional interventions are administered. Metabolomic approaches exhibit great potential for the study of arginine metabolism and determination of the relationship among arginine supplementation, health, and disease. This study would contribute in determining the effects of metabolic modifiers and modifying nutritional requirements to develop improved nutritional supplements for growth and health. The purpose of this study were to investigate the effects of arginine supplementation on growth performance and urinary composition of rats by using ^1^H-NMR spectroscopy and chemometrics.

## 2. Results

### 2.1. Effect of Arginine Supplementation on Growth Performance

The mean daily body weight gain of arginine supplementation was higher than that of the control group (22.03%) (*p* < 0.05, Table 1).

### 2.2. ^1^H-NMR Spectra of Urine Samples

**Figure 1:**
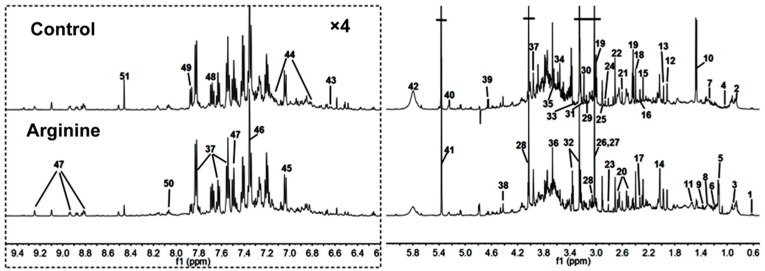
Typical one-dimensional ^1^H-NMR spectra obtained from representative rat urine samples of the control and arginine groups. The region of δ 6.2 to δ 9.5 was magnified four times compared with the corresponding region of δ 0.7 to δ 6.2 for clarity. A total of 51 metabolites were assigned. The chemical shifts and peak multiplicities of these metabolites are shown in Table 2.

Figure 1 shows typical ^1^H-NMR spectra of urine samples from randomly selected rats in the arginine and control groups. The NMR signals were assigned to specific metabolites using the ^1^H resonances (Table 2). A total of 51 metabolites were distributed in urine. The spectra of the urine samples contained resonances from allantoin, glucose, and choline, as well as several amino acids and organic acids. Tricarboxylic acid (TCA) cycle metabolites, such as succinate and citrate, were also detected in the urine samples.

### 2.3. Multivariate Data Analysis of NMR Data

**Figure 2:**
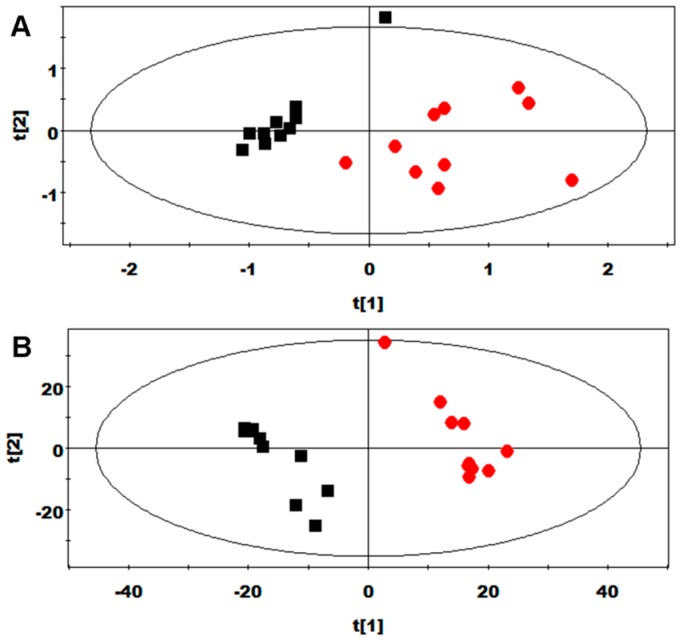
PCA score plots (*R*^2^*X* = 0.456 and *Q*^2^ = 0.141; (**A**) and PLS-DA score plots (*R*^2^*X* = 0.312, *R*^2^*Y* = 0.978, and *Q*^2^ = 0.873; (**B**) obtained through ^1^H-NMR spectra of 24 h urine samples from the arginine (red circles) and control (black squares) groups.

PCA and PLS-DA were initially performed on the urine spectral data (Figure 2). Two principal components (PCs) were calculated for the treatment groups, with 30.1% and 15.5% of the variables being explained by PC1 and PC2, respectively. The PCA results (Figure 2A) demonstrated that separations in rats from the arginine and control groups were absent in their metabolic plasma profiles. PLS-DA was then performed on the urine spectra of the arginine and control groups. The score plots (Figure 2B) highlighted two clusters that corresponded to the two groups. The metabolic profiles of both groups were compared with OPLS-DA to identify key metabolic changes in urine. 

**Figure 3:**
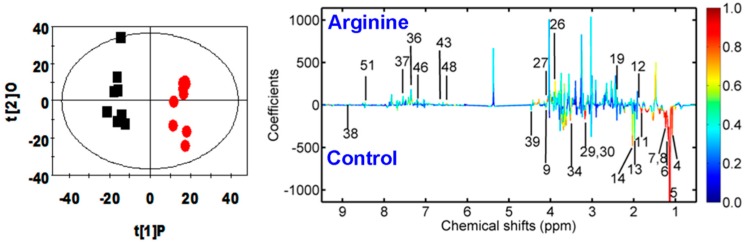
OPLS-DA scores plots (**left** panel) and the corresponding coefficient loading plots (**right** panel) obtained from the ^1^H-NMR spectra of 24 h urine samples from the control (black squares) and arginine (red circles) groups (*R*^2^*X* = 0.312, *Q*^2^ = 0.529). Two samples from the control group were excluded because they were outside the Hotelling’s *T*^2^ ellipse on the score plot. A color scale in the coefficient plot demonstrates the significance of metabolite variation between the arginine and control groups.

Multivariate data analysis showed that arginine significantly increased the urine levels of 4-aminohippurate, acetate, creatine, creatinine, ethanolamine, formate, hippurate, homogentisate, indoxyl sulfate, and phenylacetyglycine but decreased the urine levels of acetamide, β-glucose, cirtulline, ethanol, glycine, isobutyrate, lactate, mmalonate, methymalonate, *N*-acetylglutamate, *N*-methylnicotinamide, and propionate (Figure 3 and Table 3).

## 3. Discussion

Arginine supplementation can modulate body metabolism, and enhance growth performance in animals. We believe this paper is the first report on urinary metabolite profiles after arginine supplementation under non-oxidative stress conditions. Previous experiments have confirmed that 1% arginine supplementation can increase growth performance of animals [16,29,30,31]. Thus, a dose of 1% arginine supplementation was used. In this experiment, the daily bodyweight gain of arginine were higher than that of contol group. This is consistent with the previous results [16,29,30,31]. Collectively, arginine supplementation can enhance growth performance in rats. The growth performance is in connection with body metabolism modulated by arginine administration.

Arginine supplementation can alter energy metabolism and decrease urinary glucose levels. Glucose is a major substrate that offers energy for animal growth and development. Similarly, a previous study indicated that arginine can reduce plasma glucose levels [12]. Lactate is a glycolytic end product associated with energy metabolism. Decreased lactate concentration was observed in the urine sample of the arginine group, which is in accordance with a previous study, in which arginine decreased muscle lactate content in growing-finishing pigs [16]. Decreased urinary lactate and glucose levels implied the modification of carbohydrate and energy metabolism. Creatine offers energy to invertebrate muscles in the form of stored creatine phosphate. Creatine is synthesized de novo in the liver of animals from amino acids, such as arginine, glycine, and methionine. In this study, urinary creatine levels increased in the arginine group compared with that in the control group. Moreover, α-ketoglutarate, an important intermediate in the TCA cycle, increased. These findings indicated that arginine supplementation can affect energy metabolism in rats.

Arginine can change amino acid metabolism. Increased creatinine levels have been observed to be related to growth [30]. As such, creatinine has been regarded as an index of muscle mass [32], which confirmed the present findings. Arginine exhibits important functions in increasing protein synthesis, resulting in conversion of large amounts of amino acids into protein. In addition, arginine decreased urinary citrulline and *N*-acetylglutamate levels. Citrulline is an amino acid produced from ornithine and carbamoyl phosphate through a central reaction in the urea cycle. This amino acid is derived from arginine as a by-product of the reaction catalyzed by the nitric oxide synthase family. In this reaction, arginine is first oxidized into *N*-hydroxyl-arginine and then oxidized to citrulline; these processes are accompanied with the release of nitric oxide [33]. Nitric oxide can modulate self-renewal, migration, proliferation, and differation of cells. *N*-Acetylglutamate is required for the normal function of the urea cycle, and variations in *N*-acetylglutamate concentrations affect the production rate of urea and other substrates used for urea synthesis [34]. Moreover, homogentisate is considered as an intermediate of the metabolic breakdown of tyrosine and phenylalanine [35]. In this study, urinary homogentisate concentration significantly increased. These findings indicated that arginine can alter nitrogen metabolism in rats.

Arginine supplementation alters gut microbiota functions. In this study, arginine exhibited significant effects on the urinary concentrations of formate, ethanol, and SCFAs (such as isobutyrate, propionate and acetate) in rats. The levels of urine acetate and formate increased, whereas urine propionate, isobutyrate and ethanol decreased in the arginine group. These metabolites are possibly manufactured or utilized by gut microbiota. These results are in accordance with those of previous studies, in which these plasma microbiota metabolites were regulated by arginine supplementation [29]. Arginine reduced the urinary excretion of hippurate, which is produced by renal and hepatic synthesis of glycine and benzoic acid. Hippurate is produced through flavonol degradation by intestinal microorganisms [36]. Changes in the excretion of this compound implied a corresponding change in the functional metabolism of the microbiota. Changes in the urinary hippurate concentration have also been associated with variations in the distribution of intestinal microbial colonies [37]. Alterations in the gut microbial co-metabolites, such as phenylacetylglycine, confirmed the relationship between disturbance of gut microbiota and arginine exposure. The action of gut microbiota causes the transformation of phenylalanine to phenylacetate, which is then conjugated with glycine to produce phenylacetylglycine [37]. Mammalian metabolism is significantly affected by the complicated gut microbiota. The introduction of arginine into the mammalian system may displace baseline mammalian-to-microbial behavior, thereby leading to the disruption of the microbial populations and eventually affecting metabolism. In this study, biomarkers, such as α-keto-glutarate, *N*-acetylglutamate, homogentisate, hippurate, and phenylacetylglycine, were detected in urine. These metabolites were not present in the plasma metabolome of pigs [29,30]. Moreover, the results of metabolites such as bile acids, creatine, citrate, acetate, formate, hippurate, and phenylacetylglycine under non-oxidative stress (arginine group vs. control group) are different from those under oxidative stress (arginine + diquat group vs. diquat group) [31], which suggest urinary metabolome variations are different under oxidative stress and non-oxidative stress. Furthermore, a novel and unexpected finding from this study is that urinary concentrations of acetate, formate, hippurate, phenylacetylglycine were increased under non-oxidative stress (arginine group vs. control group), not oxidative stress (arginine + diquat group vs. diquat group) [31]. These results suggest the effects of arginine in the microbiota metabolites are more evident under non-oxidative stress than oxidative stress condition. The reason for this difference is unclear and needs further study in the future.

## 4. Materials and Methods

### 4.1. Animal Experiments and Sample Collection

The animal experiments were approved by the Animal Care and Use Committee of Sichuan Agricultural University and performed in line with the Chinese guidelines for animal welfare and experimental protocol. Twenty 8-week-old female Sprague-Dawley rats weighing 240 g to 270 g were placed in individual metabolic cages. After acclimatization for 2 weeks, the rats were randomly assigned to two dietary groups, with ten rats in each group. The rats were fed basal diet (Beijing Jiakangyuan Technology Development Co., Ltd., Beijing, China) supplemented with 0% (control) or 1% arginine for 28 days. The bodyweight of each rat was determined once a week. The daily feed intake of the rats was also recorded. The rats were allowed free access to food and drinking water. Temperatures between 22 °C and 25 °C, a cycle of 12 h light/12 h dark, and humidity ranging from 50% to 70% were maintained for the duration of the study. Clinical observations were performed during the whole experimental period. Urine samples were collected in ice-cooled vessels containing 30 μL of sodium azide solution (1.0% *w*/*v*) from day 27 to day 28 of the treatment period (24 h). All urine samples were stored at −80 °C prior to NMR analysis. The dosage selected for this study was on the basis of the results of a previous experiment [16,29,30,31].

### 4.2. Sample Preparation and NMR Spectroscopy

Urine samples (550 μL) were mixed with 55 μL of phosphate buffer (1.5 M NaH_2_PO_4_/K_2_HPO_4_; pH 7.4; 100% *v*/*v* D_2_O) with 0.1% NaN_3_ as bacterial growth inhibitor and 5.0 mM 2,2-dimethyl-2-silapentane-5-sulfonate-*d*_6_ (DSS) as chemical shift reference (δ 0.00 ppm). After centrifugation at 4 °C and 12,000 rpm for 10 min, the supernatant was transferred into 5 mm NMR tubes for subsequent NMR analysis. The proton NMR spectra of the urine samples were acquired at 300 K with a Bruker Avance II 600 MHz spectrometer (Bruker Biospin, Rheinstetten, Germany; operating at 600.13 MHz for ^1^H) equipped with a broadband-observe probe. A standard water-suppressed one-dimensional NMR spectrum was derived from urine by using the first increment of the gradient-selected NOESY pulse sequence (recycle delay–90°–*t*_1_–90°–*t*_m_–90°–acquire data) with a recycle delay of 2 s, *t*_1_ of 3 μs, mixing time (*t*_m_) of 100 ms, and 90° pulse length of 13.70 μs. A total of 128 transients were collected into 49,178 data points by using a spectral width of 9590 Hz and an acquisition time of 2.56 s. Metabolites were assigned based on chemical shifts, coupling constants, and relative intensities as described in previous reports [38,39,40] and additional ^1^H-^1^H correlation spectroscopy and ^1^H-^1^H total correlation spectroscopy were recorded for selected samples (data not shown).

### 4.3. NMR Spectroscopic Processes and Analyses

An exponential window function with a 1 Hz line-broadening factor was applied to the free induction decay before Fourier transformation. All the ^1^H-NMR spectra were manually corrected for phase and baseline distortions with the Mestrenova 8.1.2 software (Mestrelab Research S.L., Santiago de Compostela, Spain). The urinary spectral region from δ 0.5 to δ 9.5 was decreased into small bins of 0.005 ppm with the Mestrenova 8.1.2 software. Urine chemical shifts were referenced to the peak of DSS at δ 0.00. Chemical shifts for urinary citrate were manually corrected because the signals contained large inter-sample variations. The H_2_O signals and the regional urea signals were carefully excluded to avoid any contributions of urea and H_2_O to the intergroup differentiations and obtain endogenous metabolite changes induced by the treatment. The excluded regions in the urine spectra contained δ 4.50 to δ 5.30 for H_2_O and δ 5.5 to δ 6.0 for urea.

Each integral region was normalized to the total sum of the spectral integral regions for each spectrum to compensate for sample concentration differences before pattern recognition analysis. Multivariate data analysis was carried out on the normalized NMR data sets with the software package SIMCA-P+ (version 11.0, Umetrics, Umeå, Sweden). Principal component analysis (PCA) was performed on the mean-centered NMR data to generate an overview and identify potential outliers. Results were observed in the form of score plots, in which each point represented an individual sample, and loading plots, in which each coordinate represented one NMR spectral region. Projection to latent structure-discriminant analysis (PLS-DA) and orthogonal projection to latent structure-discriminant analysis (OPLS-DA) were conducted, with unit-variance scaled data as the X-matrix and class information as the Y-matrix [41]. The quality of the model was monitored by such model parameters, namely, *R*^2^*X* for the total explained variation and *Q*^2^ for the model predictability. The models were validated by two methods: a seven-fold cross validation method and a permutation test [42,43]. The model was interpreted using back-transformation of the loadings, with incorporated color-coded coefficient values (*r*) (Mathworks version 7.1, Mathworks Inc., Nattick, MA, USA) to reveal significantly altered metabolites responsible for the differentiation [43]. In short, each back-scaled loading is plotted as a function of the respective chemical shift with a color code, which demonstrates the weights of the discriminatory variables. In this study, appropriate correlation coefficients were used as cutoff values (depending on the number of animals used for each group) for statistical significance test based on discrimination significance (*p* < 0.05). Coefficients were determined using the Pearson’s product-moment correlation coefficient. In the loading plots, the warm-colored (e.g., red) variables correspond to metabolites that differed between classes, whereas cold-colored (e.g., blue) variables correspond to the absence of differences between classes.

## 5. Conclusions

Metabolomics is an effective approach used to discover biomarkers by analyzing global changes in the metabolic profiles of rats. The effects of arginine supplementation-supporting growth may be exerted by modulating the pathways referring to energy metabolism, amino acid metabolism, and gut microbiota metabolism. For the first time, we report a comprehensive analysis of the urinary metabolic patterns of arginine supplementation under non-oxidative stress. This study would contribute to further understanding of the mechanisms underlying the physiological functions of arginine.

## Figures and Tables

**Table 1:** Effects of arginine supplementation on body weight gain and food intake of rats (*n* = 10).

Parameters	Control	Arginine	SEM	*p* Value
Mean daily body weight gain (g)	2.95	3.60	0.16	0.03
Mean daily food intake (g)	21.95	23.91	0.59	0.14
Food intake/body weight gain ratio	8.86	7.19	1.04	0.25

**Table 2:** ^1^H-NMR data of metabolites in rat urine.

Keys	Metabolites	Moieties	δ ^1^H (ppm) and Multiplicity
1	Bile acids	CH_3_	0.64 (m), 0.75 (m)
2	α-Hydroxy-iso-valerate	δCH_3_, CH_3_	0.83 (d), 0.97 (d)
3	α-Hydroxybutyrate	CH_3_	0.89 (t)
4	Propionate	CH_3_	1.06 (t)
5	Isobutyrate	CH_3_	1.13 (d)
6	Ethanol	CH_3_	1.19 (t)
7	Methylmalonate	CH_3_, CH	1.25 (d), 3.75 (m)
8	α-Hydroxy-*n*-valerate	CH_3_, γCH_2_	0.89 (t), 1.31 (m)
9	Lactate	αCH, βCH_3_	4.14 (q), 1.33 (d)
10	Alanine	αCH, βCH_3_	3.77 (q), 1.47 (d)
11	Citrulline	γCH_2_, βCH_2_	1.56 (m), 1.82 (m)
12	Acetate	CH_3_	1.92 (s)
13	Acetamide	CH_3_	1.99 (s)
14	*N*-Acetylglutamate	βCH_2_, γCH_2_, CH_3_	2.06 (m), 1.87 (m), 2.03 (s)
15	Acetone	CH_3_	2.24 (s)
16	Acetoacetate	CH_3_	2.28 (s)
17	Pyruvate	CH_3_	2.33 (s)
18	Succinate	CH_2_	2.40 (s)
19	α-Ketoglutarate	βCH_2_, γCH_2_	2.45 (t), 3.01 (t)
20	Citrate	CH_2_	2.54 (d), 2.68 (d)
21	Methylamine	CH_3_	2.61 (s)
22	Dimethylamine	CH_3_	2.71 (s)
23	Methylguanidine	CH_3_	2.81 (s)
24	Trimethylamine	CH_3_	2.88 (s)
25	Dimethylglycine	CH_3_	2.93 (s)
26	Creatine	CH_3_, CH_2_	3.04 (s), 3.93 (s)
27	Creatinine	CH_3_, CH_2_	3.04 (s), 4.05 (s)
28	Ornithine	CH_2_	3.06 (t)
29	Ethanolamine	CH_2_	3.11 (t)
30	Malonate	CH_2_	3.15 (s)
31	Choline	OCH_2_, NCH_2_, N(CH_3_)_3_	4.07 (t), 3.53 (t), 3.21 (s)
32	Taurine	-CH_2_-S, -CH_2_-NH_2_	3.27 (t), 3.43 (t)
33	TMAO ^a^	CH_3_	3.27 (s)
34	Glycine	CH_2_	3.57 (s)
35	Sarcosine	CH_2_	3.6 (s)
36	Phenylacetyglycine	2,6-CH, 3,5-CH, 7-CH, 10-CH	7.30 (t), 7.36 (m), 7.42 (m), 3.67 (s)
37	Hippurate	CH_2_, 3,5-CH, 4-CH, 2,6-CH	3.97 (d), 7.55 (t), 7.63 (t), 7.84 (d)
38	*N*-Methylnicotinamide	CH_3_, 5-CH, 4-CH, 6-CH, CH_2_	4.42 (s), 8.21 (d), 8.87 (d), 8.93 (d), 9.24 (s)
39	β-Glucose	1-CH, 2-CH, 3-CH, 4-CH, 5-CH, 6-CH	4.47 (d), 3.25 (dd), 3.49 (t), 3.41 (dd), 3.46 (m), 3.73 (dd), 3.90 (dd)
40	α-Glucose	1-CH, 2-CH, 3-CH, 4-CH, 5-CH, 6-CH	5.24 (d), 3.54 (dd), 3.71 (dd), 3.42 (dd), 3.84 (m), 3.78 (m)
41	Allantoin	CH	5.39 (s)
42	Urea	NH_2_	5.82 (s)
43	Homogentisate	6-CH, 5-CH	6.67 (d), 6.82 (d),
44	*p*-Hydroxyphenylacetate	6-CH, 2-CH, 3,5-CH	3.6 (s), 6.85 (d), 7.15 (d)
45	*m*-Hydroxyphenylacetate	6-CH, 4-CH, 3-CH	6.92 (m), 7.04 (d), 7.26 (t)
46	Indoxyl sulfate	4-CH, 5-CH, 6-CH, 7-CH, CH	7.51 (m), 7.22 (m), 7.28 (m), 7.71 (m), 7.37 (s)
47	Nicotinamide	2-CH, 4-CH, 5-CH, 6-CH	8.94 (d), 8.61 (dd), 8.25 (m), 7.5 (dd)
48	4-Aminohippurate	CH_2_, CH	7.6 (d), 6.8 (d), 3.9 (d)
49	Benzoate	2,6-CH, 3,5-CH, 4-CH	7.87 (d),7.49 (dd), 7.56 (t)
50	Trigonelline	2-CH, 4-CH, 6-CH, 5-CH, CH_3_	9.09 (s), 8.85 (m), 8.81 (dd), 8.07 (m), 4.44 (s)
51	Formate	CH	8.46 (s)

^a^ TMAO, trimethylamine-*N*-oxide; s, singlet; d, doublet; t, triplet; q, quartet; dd, doublet of doublets; m, multiplet.

**Table 3:** OPLS-DA coefficients obtained from the NMR data of urine metabolites derived from the (A) control and (B) arginine groups.

Metabolite	OPLS-DA Coefficient (r) ^a^	*p* Value ^b^
	B (vs. A)	B (vs. A)
4-Aminohippurate (48)	0.798	<0.05
Acetamide (13)	−0.661	<0.05
Acetate (12)	0.760	<0.05
β-Glucose (39)	−0.603	<0.05
Citrulline (11)	−0.889	<0.05
Creatine (26)	0.794	<0.05
Creatinine (27)	0.915	<0.05
Ethanol (6)	−0.932	<0.05
Ethanolamine (29)	0.618	<0.05
Formate (51)	0.660	<0.05
Glycine (34)	−0.832	<0.05
Hippurate (37)	0.632	<0.05
Homogentisate (43)	0.654	<0.05
Indoxyl sulfate (46)	0.742	<0.05
Isobutyrate (5)	−0.871	<0.05
Lactate (9)	−0.715	<0.05
Malonate (30)	−0.716	<0.05
Methymalonate (7)	−0.953	<0.05
*N*-Acetylglutamate (14)	−0.855	<0.05
*N*-Methylnicotinamide (38)	−0.642	<0.05
Phenylacetyglycine (36)	0.650	<0.05
Propionate (4)	−0.825	<0.05
α-Hydroxy-*n*-valerate (8)	−0.748	<0.05
α-Ketoglutarate (19)	0.665	<0.05

^a^ Metabolite keys are shown in Table 2. Correlation coefficients were obtained from the OPLS-DA results, with the positive and negative signs suggesting the positive and negative correlation, respectively, with the concentrations. The correlation coefficient of |r| higher than 0.602 represents the cutoff value; ^b^ Normalized integral of metabolites in the spectrum (normalized to 100). Integrals of the altered metabolites were analyzed statistically using one-way analysis of variance (ANOVA) of SPSS 16.0 software (SPSS Inc., Chicago, IL, USA). *p* values are significant at the <0.05 level.
